# Strain Matching of Seasonal Influenza Vaccines and Emergence of Neuraminidase Inhibitor Resistance in China from 2015 to 2025

**DOI:** 10.3390/vaccines14070586

**Published:** 2026-06-30

**Authors:** Peiqing He, Junhao Luo, Siyu Pu, Simin Cui, Haijun Zhu, Wenfei Zhu, Rongbao Gao

**Affiliations:** 1 NHC Key Laboratory of Biosafety, NHC Key Laboratory of Medical Virology and Viral Diseases, National Institute for Viral Disease Control and Prevention, Chinese Center for Disease Control and Prevention, Beijing 102206, China; becky_hpq@163.com (P.H.); luojh0920@foxmail.com (J.L.); wananpsy@163.com (S.P.); 17791366202@163.com (S.C.); zhuwf@ivdc.chinacdc.cn (W.Z.); 2 Department of Health Inspection and Quarantine, School of Public Health, Public Health College, Anhui Medical University, Hefei 230032, China; 18225855528@163.com

**Keywords:** influenza virus, neuraminidase resistance, antigenic matching

## Abstract

**Background**: Influenza remains a major global public health threat, and vaccination is one of the most effective preventive measures. However, frequent antigenic drift and occasional antigenic shift, along with the lead time required for vaccine development and regional differences in the evolution of circulating strains, may lead to mismatches between WHO-recommended vaccine strains and circulating viruses. In addition, antiviral resistance further complicates precise influenza prevention and control. **Objectives**: This study aimed to evaluate the concordance of vaccine strains with circulating influenza viruses and the emergence of neuraminidase inhibitor (NAI) resistance in China. **Methods**: Data on antigenic characterization and antiviral susceptibility testing were extracted from weekly influenza surveillance reports published by the Chinese National Influenza Center from 2015 to 2025. Viral evolution, substitutions at key antigenic sites, and resistance-associated mutations were further examined based on sequences of circulating influenza viruses in China. **Results**: The overall vaccine match rates were 95.72% (95% CI: 94.02–97.43%) for A(H1N1)pdm09, 58.96% (95% CI: 54.93–62.96%) for A(H3N2), 64.45% (95% CI: 59.49–69.41%) for B/Victoria, and 95.19% (95% CI: 91.32–99.05%) for B/Yamagata in China during the 2015–2025 influenza seasons, with marked year-to-year fluctuations observed particularly for A(H3N2) and B/Victoria. The vaccine matching for cell-based A(H3N2) (70.41%, 95% CI: 65.04–75.77%) vaccine reference strains was significantly higher than that for egg-based A(H3N2) (48.09%, 95% CI: 42.63–53.55%) vaccine reference strains. Sequence analysis indicated that circulating A(H3N2) viruses showed the greatest genetic divergence from the matched egg-based vaccine strains (2.71%, 95% CI: 2.66–2.75%). Phenotypic NAI resistance was detected only in A(H1N1)pdm09 viruses, with resistance rates of 0.18% (95% CI: 0.07–0.45%) in 2023, 3.47% (95% CI: 2.63–4.57%) in 2024, and 3.01% (95% CI: 2.46–3.68%) in 2025. Neuraminidase (NA) sequence analysis showed that the key NAI resistance-associated substitution H274Y has been detected in A(H1N1)pdm09 viruses since 2015, at relatively high frequencies observed during 2015–2018. The mutation re-emerged in 2023 and presented increase trends thereafter, although no A(H1N1) pdm09 circulated during the COVID-19 pandemic. **Conclusions**: Antigenic concordance between vaccine strains and circulating A(H3N2) or B/Victoria viruses showed marked year-to-year fluctuations in China. Cell-based A(H3N2) vaccine reference strains showed higher antigenic concordance than egg-based strains, supporting further consideration of vaccine production platforms in A(H3N2)-predominant seasons. Phenotypic NAI resistance in circulating A(H1N1)pdm09 viruses was detected from 2023 onward in China, whereas resistance-associated NA substitutions had been detected earlier at the sequence level.

## 1. Introduction

Seasonal influenza is an acute respiratory infection caused by influenza viruses, which spread efficiently via respiratory droplets generated by coughing and sneezing. It is estimated to cause one billion cases each year, including 3–5 million severe cases. Influenza viruses are classified into four types: A, B, C, and D. Human seasonal epidemics are caused primarily by influenza A and influenza B viruses [1]. In China, influenza activity follows a clear latitudinal pattern, with a short but intense winter peak in high-latitude regions and semiannual peaks or year-round circulation in low- and mid-latitude regions. Influenza A(H1N1)pdm09 and B viruses are most prevalent in winter, whereas A(H3N2) is the main driver of summer epidemics in low- and mid-latitude regions [2]. Humans are generally susceptible to influenza viruses, with children and older adults at higher risk of severe disease and illness and mortality. Vaccination remains one of the most effective preventive measures. Current influenza vaccines are intended to induce neutralizing antibodies to prevent viral attachment to and entry into host cells. Nevertheless, novel viruses with antigenically distinct hemagglutinin (HA) proteins frequently emerge through antigenic drift, which is driven by the error-prone nature of viral RNA replication, or through antigenic shift, which results from gene segment reassortment among co-circulating influenza viruses [3]. Thus, emerging epidemic strains may not be effectively neutralized by pre-existing antibodies because antigenic changes can reduce antibody binding ability.

Given the frequent antigenic drift of influenza viruses, the World Health Organization (WHO) annually updates its vaccine strain recommendations for the upcoming influenza season based on global surveillance data for circulating strains. Vaccine strains are typically selected 6–8 months in advance to predict the strains likely to circulate in the upcoming season; however, the virus may still undergo new mutations during the vaccine production and vaccination cycle. The high mutability of influenza viruses [4], together with the substantial time lag in vaccine development [5], may lead to mismatches between vaccine strains and circulating strains [6]. In addition, globally recommended vaccine strains may not fully align with regional epidemic characteristics because the evolutionary lineages of circulating strains may vary across regions [7], thereby increasing the risk of mismatch and potentially reducing vaccine effectiveness at the population level, and posing challenges to precise influenza control and prevention.

Meanwhile, the circulation of antiviral-resistant influenza viruses has further increased the complexity of influenza prevention and control. Neuraminidase inhibitors (NAIs), including oseltamivir, zanamivir, and peramivir, remain important antiviral options for influenza treatment, especially for patients at high risk of severe disease. Influenza viruses evolve rapidly because their RNA-dependent RNA polymerase lacks proofreading activity, and inappropriate or prolonged antiviral use may further increase selective pressure for the emergence of resistant variants [8]. NAI-resistant strains may show reduced susceptibility to antiviral drugs and compromise treatment effectiveness. For example, the NA H274Y substitution in seasonal A(H1N1)pdm09 viruses and the NA R292K substitution in A(H3N2) viruses have been widely reported to confer resistance to oseltamivir [9,10]. Because antiviral-resistant strains are difficult to distinguish from susceptible strains based on clinical presentation alone [11], laboratory-based surveillance, including phenotypic susceptibility testing and gene sequencing, is required for their identification. However, limited testing capacity in primary-level public health laboratories may hinder early detection and epidemiological tracking of NAI-resistant strains, thereby hindering the timely implementation of targeted control measures. Therefore, continuous monitoring of NAI resistance and related NA mutations is important for guiding antiviral use, evaluating resistance risk, and improving influenza prevention and control strategies.

In this study, to assess the concordance between vaccine strains and circulating strains as well as the emergence of neuraminidase inhibitor (NAI) resistance in China, we analyzed the antigenic characterization and antiviral susceptibility data from weekly influenza surveillance reports from 2015 to 2025, and examined viral evolution, changes at key antigenic sites, and resistance-associated mutations in sequences from circulating viruses in China. These findings may support the refinement of influenza prevention and control strategies.

## 2. Materials and Methods

### 2.1. Data Source

#### 2.1.1. Data of Vaccine Antigenic Analysis

The summary data on influenza vaccine antigenic analyses used in this study were obtained from the weekly influenza surveillance reports published on the website of the Chinese National Influenza Center (CNIC), Chinese Center for Disease Control and Prevention (https://ivdc.chinacdc.cn/cnic/, accessed on 6 March 2026). We extracted antigenic surveillance data on influenza strains circulating in China from week 41 of 2015 through week 40 of 2025, including influenza A(H1N1)pdm09, A(H3N2), B/Victoria, and B/Yamagata, focusing on their match to the seasonal vaccine strains recommended by the World Health Organization and the proportion of low-reacting strains. The number of antigenically characterized viruses and vaccine-like viruses for each subtype/lineage and influenza season is summarized in Table S1. In this study, each influenza season was defined as the period from week 41 of one year to week 40 of the following year.

Vaccine antigenic match was assessed according to the antigenic characterization results reported by CNIC. Viruses reported as antigenically similar to the corresponding WHO-recommended vaccine reference strain were classified as vaccine-matched strains. Low-reacting strains were defined as viruses showing an ≥8-fold reduction in HI titers compared with the corresponding vaccine strains [12]. This definition was applied consistently across all influenza subtypes/lineages and study season.

For each influenza season, vaccine-like viruses referred to viruses that were reported as antigenically similar to the corresponding WHO-recommended seasonal vaccine or reference strain. The vaccine match rate was calculated as follows:

Vaccine match rate (%) = number of vaccine-like viruses within the influenza season/total number of antigenically characterized viruses within the influenza season × 100.

#### 2.1.2. Data of Viral Gene Sequence

The HA and NA gene sequences of influenza viruses were downloaded from the Global Initiative on Sharing All Influenza Data (GISAID, https://www.gisaid.org/, accessed on 6 March 2026). We included sequences collected between 1 October 2015 and 30 September 2025 to match the reporting period of the National Influenza Center weekly surveillance reports, sampled in mainland China only (excluding Hong Kong, Macao, and Taiwan), and with at least 95% sequence completeness. All sequences were obtained and used in accordance with GISAID requirements, and their sources and related metadata were appropriately acknowledged.

Sequences with incomplete collection dates, sampling locations, or subtype/lineage annotations were excluded. After quality filtering, a total of 11,925 HA sequences, including 3078 A(H1N1)pdm09, 4319 A(H3N2), 3962 B/Victoria, and 566 B/Yamagata sequences, were retained for sequence-based analyses. In addition, 3032 A(H1N1)pdm09 NA sequences were retained for resistance-associated mutation analysis. The numbers of HA and NA sequences included for each subtype/lineage are summarized in Supplementary Table S2. HA sequences were used for analyses of HA1 genetic distance, dN/dS ratios, and antigenic-site substitutions, whereas NA sequences were used to screen mutations at antiviral resistance-associated sites.

### 2.2. Methods

#### 2.2.1. Analysis of Vaccine Antigenicity Matching

Data from weekly influenza surveillance reports of CNIC were analyzed using descriptive epidemiologic methods to assess the proportion of circulating A(H1N1)pdm09, A(H3N2), and influenza B strains that were antigenically similar to the corresponding vaccine strains, including both egg-based and cell-based (only A(H3N2) involved) vaccine reference strains. We then compared the degree of vaccine–strain matching across influenza subtypes. In addition, HA sequence data were subsequently examined to explore genetic changes potentially associated with antigenic mismatch, particularly amino acid substitutions at key antigenic sites in the HA protein.

#### 2.2.2. Analysis of Neuraminidase Inhibitor Resistance

This analysis focused specifically on resistance to neuraminidase inhibitors (NAIs) among influenza A(H1N1)pdm09 viruses circulating in China. Weekly data on NAI susceptibility testing were extracted from CNIC influenza surveillance reports. Descriptive epidemiologic analyses were performed to summarize temporal changes in NAI resistance among A(H1N1)pdm09 viruses across surveillance weeks and influenza seasons. The NAI resistance rate was calculated as the number of resistant A(H1N1)pdm09 viruses divided by the total number of A(H1N1)pdm09 viruses tested for NAI susceptibility, multiplied by 100. Only A(H1N1)pdm09 viruses with available NAI susceptibility testing results were included in the resistance-rate calculation.

#### 2.2.3. HA Gene Sequence Analysis

HA gene sequences of circulating viruses and the corresponding WHO-recommended vaccine reference strains were aligned using MEGA12 software (Version 12.0.11.). The HA1 region was extracted for subsequent analyses. For each subtype/lineage and influenza season, circulating strains were compared with the corresponding vaccine reference strain. Pairwise amino acid distances in the HA1 region were calculated using the p-distance model in the Distance Estimation module of MEGA12, with uniform rates among sites and pairwise deletion for gaps or missing data.

Nucleotide sequences of the HA1 region were used to estimate nonsynonymous and synonymous substitutions. The dN/dS ratios were estimated using the Nei–Gojobori method (Proportion) in MEGA12, with analytical variance estimation and pairwise deletion for gaps or missing data. Amino acid residues at defined antigenic sites were compared between circulating strains and vaccine reference strains to identify antigenic-site substitutions.

Antigenic sites were selected according to previously published definitions for each influenza subtype/lineage. The antigenic sites included Sa, Sb, Ca1, Ca2, and Cb for A(H1N1)pdm09 [13]; sites A–E for A(H3N2) [14]; and the 120-loop, 150-loop, 160-loop, and 190-helix regions for influenza B viruses [15]. Amino acid positions were numbered according to subtype/lineage-specific HA reference strains and corresponding HA numbering conventions. Specifically, A(H1N1)pdm09 antigenic sites were numbered according to H1 HA numbering, A(H3N2) antigenic sites according to H3 HA numbering, and B/Victoria and B/Yamagata antigenic sites according to influenza B HA numbering. For each influenza season, amino acid residues at defined antigenic sites in circulating strains were compared with those of the corresponding WHO-recommended vaccine strain. The frequency of antigenic-site substitutions was calculated as the number of circulating HA sequences showing amino acid differences from the corresponding vaccine strain at a given antigenic site divided by the total number of HA sequences analyzed for the corresponding subtype/lineage and influenza season, multiplied by 100.

#### 2.2.4. Analysis of Resistance-Associated Amino Acid Mutations in NA

NA gene sequences of A(H1N1)pdm09 viruses retrieved from GISAID were aligned using MEGA12 software and translated into amino acid sequences. Amino acid substitutions at previously reported neuraminidase inhibitor (NAI) resistance-associated sites were screened, with particular attention paid to substitutions related to reduced susceptibility to oseltamivir. The frequency of each resistance-associated substitution was calculated as the number of A(H1N1)pdm09 NA sequences carrying the substitution divided by the total number of A(H1N1)pdm09 NA sequences analyzed for the corresponding influenza season, multiplied by 100. These analyses were used to describe temporal changes in NAI resistance-associated substitutions at the genetic level and to provide supporting information for antiviral resistance surveillance.

#### 2.2.5. Statistical Analysis

For graphical presentation, vaccine match rates were expressed as mean ± SD. Means and 95% confidence intervals (CIs) were also calculated for descriptive statistical analyses. For NAI resistance rates, 95% confidence intervals were calculated based on binomial proportions using the Wilson score method, with the number of resistant viruses as the numerator and the total number of viruses tested for NAI susceptibility as the denominator.

One-way ANOVA analysis was used for group comparisons of vaccine match rates, NAI resistance rates, HA1 amino acid distances, and dN/dS ratios. Pearson’s chi-square test was used only for comparisons of mutation frequencies among major NAI resistance-associated substitutions in A(H1N1)pdm09 viruses. *p* value < 0.05 was considered statistically significant. Statistical analyses were performed using IBM SPSS Statistics version 27.0 (IBM Corp., Armonk, NY, USA). All figures were generated using GraphPad Prism version 10.5.0 (GraphPad Software, San Diego, CA, USA).

## 3. Results

### 3.1. The Concordance Between Vaccine Strains and Circulating Strains

**Figure 1:**
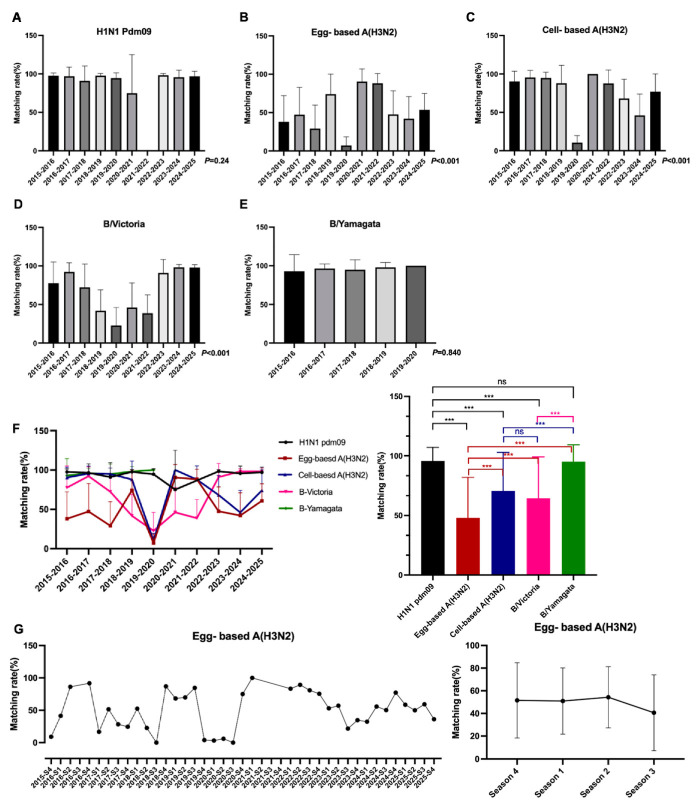
Temporal variation in antigenic matching between seasonal influenza viruses and WHO-recommended vaccine strains, 2015–2025. (**A**–**E**) Annual antigenic match rates (%) between circulating A(H1N1)pdm09 (**A**), egg-based A(H3N2) (**B**), cell-based A(H3N2) (**C**), B/Victoria (**D**), and B/Yamagata (**E**) viruses and the corresponding WHO-recommended vaccine strains during 2015–2025. Bars represent mean vaccine match rates, and error bars indicate standard deviations (SD). *p* values were calculated using one-way ANOVA to compare seasonal vaccine match rates among influenza seasons within each subtype/lineage. The number of weekly observations included in each season is provided in Table S1. (**F**) Temporal trends in antigenic match rates across groups and statistical comparisons among groups. (One-way ANOVA, Dunnett’s T3 multiple-comparison test; ***, *p* < 0.001; ns, not significant.) (**G**) Seasonal fluctuations in egg-based A(H3N2) match rates by surveillance week, 2015–2025.

Since week 41 of 2015, the vaccine match rate for circulating A(H1N1)pdm09 viruses exceeded 90% in each influenza season, except for the 2021–2022 season, when A(H1N1)pdm09 was not detected (Figure 1A). The match rate for influenza A(H3N2) exhibited considerable variability, reaching as low as 7.18% (95% CI: 1.41–12.96%) for the egg-based vaccine strain during the 2019–2020 season, which was markedly lower than those observed for other subtypes/lineages (Figure 1B). The cell-based vaccine strain increased the yearly match rate of H3N2; nonetheless, the match rate remained at a low level of 10.56% (95% CI: 5.32–15.79%) in the 2019–2020 season (Figure 1C). For influenza B viruses, the B/Victoria match rate remained above 70% from 2015 to 2018, with a peak of approximately 90% in the 2016–2017 season. It then declined and fluctuated markedly during 2018–2022, reaching its lowest level of approximately 20% in the 2019–2020 season, before rebounding to above 90% during the 2022–2025 seasons (Figure 1D). In contrast to B/Victoria, the vaccine match rate for the B/Yamagata lineage remained above 95%. The B/Yamagata lineage has not been detected in routine global influenza surveillance since 2020 (Figure 1E), and its current epidemiological status remains uncertain [16]. Overall match rates were similar for the B/Yamagata lineage (95.19%, 95% CI: 91.32–99.05%) and A(H1N1)pdm09 (95.72%, 95% CI: 94.02–97.43%), and both were higher than that for the B/Victoria lineage (64.45%, 95% CI: 59.49–69.41%). The B/Victoria lineage had a significantly higher match rate than egg-based A(H3N2) vaccine strains (48.09%, 95% CI: 42.63–53.55%) but did not differ significantly from cell-based A(H3N2) vaccine strains (70.41%, 95% CI: 65.04–75.77%). Cell-based A(H3N2) vaccine strains showed a significantly higher match rate than egg-based A(H3N2) vaccine strains (Figure 1F). Analysis of seasonal trends in the match rate for egg-based A(H3N2) vaccine strains showed no clear seasonal pattern in the match between circulating strains and vaccine strains, suggesting that these variations were difficult to predict (Figure 1G). Furthermore, no significant interannual difference was detected for A(H1N1)pdm09 (*p* = 0.240) or B/Yamagata (*p* = 0.840), whereas the annual match rates differed markedly for egg-based A(H3N2), cell-based A(H3N2), or B/Victoria (all *p* < 0.01), suggesting that A(H3N2) and B/Victoria should be given priority in vaccine strain selection.

### 3.2. Molecular Evolution of the HA1 Gene and Its Association with Antigenic Matching in Influenza Viruses

**Figure 2:**
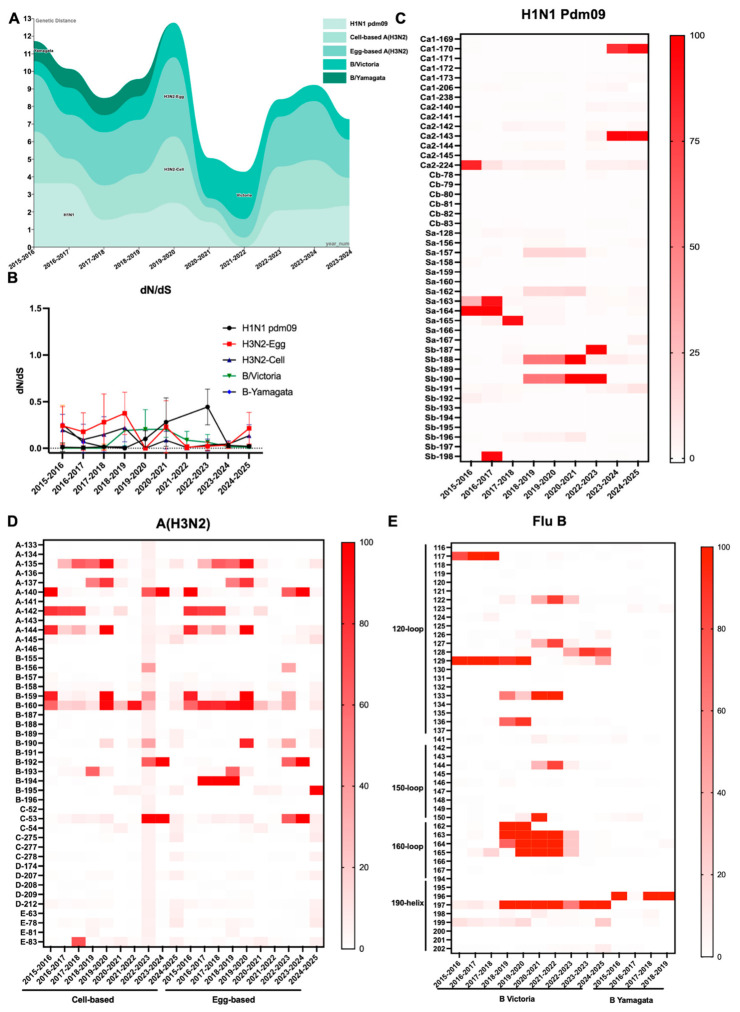
Evolutionary pressure and mutation frequencies at antigenic sites in influenza viruses during 2015–2025. (**A**) Stacked area plot showing temporal changes in HA1 genetic distances for influenza A(H1N1)pdm09, A(H3N2), B/Victoria, and B/Yamagata viruses. The plot is intended to visualize subtype/lineage-specific trends in one panel and does not represent additive or cumulative genetic distances. (**B**) Changes in the mean dN/dS ratios of different influenza virus subtypes/lineages, including A(H1N1)pdm09, Egg-based A(H3N2), Cell-based A(H3N2), B/Victoria, and B/Yamagata, across influenza seasons during 2015–2025. Error bars indicate standard deviations. (**C**–**E**) Heatmaps show the frequencies (%) of mutations at known HA1 antigenic sites relative to the corresponding vaccine strains across influenza seasons for A(H1N1) pdm09 (**C**), A(H3N2) (**D**), and influenza B viruses (**E**). Colors range from light to dark with increasing mutation frequency (0–100%), allowing temporal variation at antigenic sites to be visualized directly.

To understand the influence of viral evolutionary dynamics on vaccine concordance, we analyzed HA1 genetic distances between circulating strains and the corresponding recommended vaccine strains, dN/dS ratios, and site-specific mutations within antigenic regions. Circulating A(H3N2) strains and B/Victoria viruses exhibited more rapid antigenic drift than A(H1N1)pdm09 and B/Yamagata viruses in China from October 2015 to September 2025. In the 2019–2020 season, the HA1 genetic distance for A(H3N2) reached a peak of 4.14%. In comparison, A(H1N1)pdm09 and the B/Yamagata lineage showed lower genetic distances at 2.28% and 1.02%, respectively. In the 2020–2022 period, the HA1 genetic distance for A(H3N2) decreased to 1.05%, whereas that for the B/Victoria lineage increased to 2.46%. These divergent trends may have been influenced by changes in influenza circulation during the COVID-19 pandemic, when public health restrictions substantially reduced viral transmission [17]. During this period, the B/Victoria lineage was the predominant circulating lineage in China, whereas A(H3N2) was markedly suppressed in the early phase of this period and did not re-emerge until 2022 [18,19]. Together, these findings suggest that A(H3N2) and B/Victoria may continue to pose challenges for future vaccine strain matching (Figure 2A). The dN/dS values for the HA coding region were below 1.0 in all influenza subtypes and lineages during the 2015–2025 seasons, indicating overall purifying selection. Egg-based A(H3N2) showed relatively higher dN/dS values than the other groups in most seasons, with a peak in 2018–2019, suggesting greater nonsynonymous substitution pressure. However, these values do not support strong positive selection at the HA gene level. A(H1N1)pdm09, B/Victoria, and B/Yamagata maintained lower dN/dS values throughout most seasons, consistent with stronger evolutionary constraints. Cell-based A(H3N2) showed intermediate values, suggesting lower sequence divergence than egg-based A(H3N2) (Figure 2B).

Analysis of the site-specific mutation rate within the antigenic determinant region of HA1 protein showed that only occasional mutations were detected at sites Ca-224, Sa-164, Sa-165, and Sb187-190 in A(H1N1)pdm09 viruses, whereas most sites in the Cb region remained at a mutation rate of 0% throughout the study period, indicating a high degree of conservation (Figure 2C). In the A(H3N2) subtype, antigenic sites A and B showed higher levels of variability, and sites such as A-142 and B-160 remained variable across multiple epidemic seasons. By contrast, antigenic sites C, D, and E showed overall lower levels of variation and may represent potential targets for the development of broadly protective vaccines (Figure 2D). In the B/Victoria lineage, the 120-loop and 150-loop were relatively conserved among all antigenic regions. Antigenic sites 162–165 in the 160-loop formed a major variant cluster during 2018–2023, whereas in the 190-helix, only site 197 showed mutations after 2018, with the mutation rate remaining at 93.43%. In contrast, the overall mutation rate of the B/Yamagata lineage was remarkably low, and the overwhelming majority of antigenic sites remained highly conserved throughout the surveillance period, which was consistent with its stable genetic distance and the persistently high level of vaccine match (Figure 2E).

### 3.3. The Characterization and Emergence of NAI Resistance

**Figure 3:**
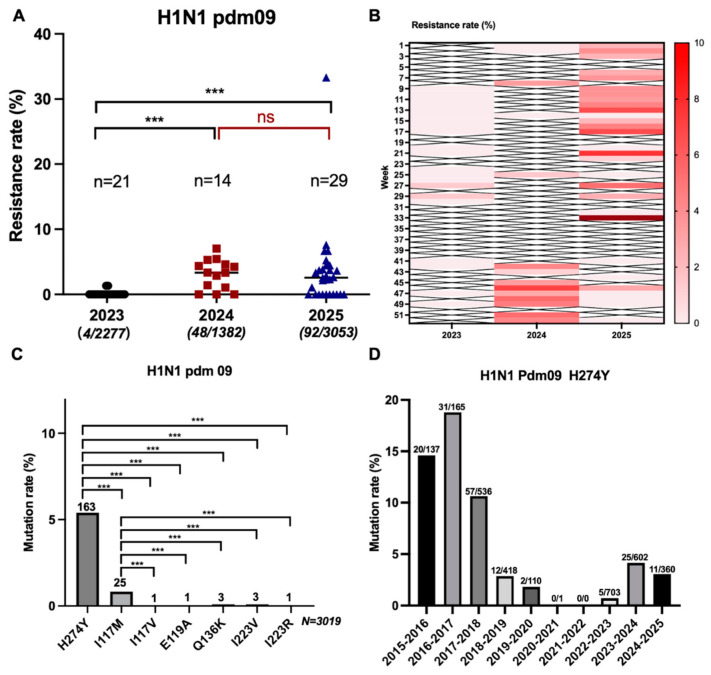
Resistance to neuraminidase inhibitors and associated mutation profiles in A(H1N1)pdm09 viruses. (**A**) Weekly resistance rates (%) of A(H1N1)pdm09 viruses to neuraminidase inhibitors from 2023 to 2025. The annual numbers of resistant/tested viruses were 4/2277 in 2023, 48/1382 in 2024, and 92/3053 in 2025. One-way ANOVA was used for annual comparisons, Dunnett’s T3 multiple-comparison test; ***, *p* < 0.001; ns, not significant. (**B**) Heatmap showing weekly neuraminidase inhibitor resistance rates from 2023 to 2025. (**C**) Mutation frequencies (%) of major neuraminidase inhibitor resistance-associated substitutions in A(H1N1)pdm09 viruses from 2015 to 2025. Pearson’s chi-square test was used for comparisons among substitutions. ***, *p* < 0.001. (**D**) Temporal trends in the frequency (%) of the H274Y mutation in A(H1N1)pdm09 viruses, 2015–2025.

Analysis of phenotypic neuraminidase inhibitor susceptibility data from CNIC weekly influenza surveillance reports showed that NAI-resistant viruses were first reported in China in 2023. Phenotypic resistance was detected only in A(H1N1)pdm09 viruses, with a rate of 0.18% (95% CI: 0.07–0.45%) in 2023, 3.47% (95% CI: 2.63–4.57%) in 2024, and 3.01% (95% CI: 2.46–3.68%) in 2025. No statistically significant difference was observed between 2024 and 2025, although an isolated high value was observed at week 32 in 2025 (Figure 3A,B). Previous reports have identified various NAI resistance-associated mutations including I117M, I117V [20], E119A [21], Q136K [22], I223V [23], I223R [24], and H274Y [25,26] in NA. H274Y represents a histidine-to-tyrosine substitution in the neuraminidase protein. Genotypic analysis of NA sequences retrieved from GISAID showed that H274Y and I117M were the most frequently detected resistance-associated substitutions in circulating A(H1N1)pdm09 viruses in China from 2015 to 2025. H274Y and I117M were detected in 5.5% and 0.62% of NA sequences, respectively, whereas I117V, E119A, Q136K, I223V, and I223R were each detected in <0.1% of sequences (Figure 3C). Temporal analysis showed that H274Y was detected at a relatively high frequency of 14.60% in the 2015–2016 season, reached a peak of 18.79% in the 2016–2017 season, and declined markedly after the 2018–2019 season. No H274Y-positive A(H1N1)pdm09 sequences were detected during the 2020–2022 seasons, whereas this substitution reappeared in 2023 and reached 3.06% in the 2024–2025 season (Figure 3D). These genotype-based mutation frequencies were calculated from publicly available GISAID NA sequences and were not directly paired with the phenotypic susceptibility data reported in CNIC weekly reports. Therefore, H274Y frequency should be interpreted as a marker of potential oseltamivir resistance rather than as a direct estimate of phenotypic NAI resistance.

## 4. Discussion

The annual selection of influenza vaccine strains is challenged by viral evolution and the time lag between strain recommendation and vaccine use [27]. Antigenic matching between vaccine strains and circulating strains is an important factor associated with vaccine effectiveness, but it does not fully determine clinical protection. In this study, antigenic mismatch was observed more often among influenza A(H3N2) and B/Victoria viruses circulating in China than for A(H1N1)pdm09 or B/Yamagata viruses, which is broadly consistent with findings reported worldwide. A meta-analysis by Tricco et al. showed that mismatch was more commonly observed in influenza A(H3N2) and B/Victoria viruses, and that vaccine effectiveness against these viruses varied more substantially [28]. An epidemiological study conducted in Hong Kong, China, during 2009–2012 reported that the seasonal drift rate for A(H3N2) reached 41.2%, with a vaccine match rate of only 20.6% for the Northern Hemisphere strain; the overall match rate for influenza B was 14.7% [29]. Surveillance data from the European Union during the 2023–2024 influenza season showed that A(H3N2) underwent rapid genetic diversification and accumulated mutations at key antigenic sites, and that 23% of circulating viruses were antigenically distinct from the vaccine strain used in that season. Vaccine effectiveness was also lower against A(H3N2) than against A(H1N1)pdm09. In comparison, A(H1N1)pdm09 remained closely antigenically matched to the vaccine strain and showed greater antigenic stability [30].

Sequence analysis showed that A(H1N1)pdm09 viruses remained genetically close to the corresponding vaccine strains throughout the study period. Only a few amino acid mutations were observed at HA1 antigenic sites, and the dN/dS ratio remained low. Collectively, these findings suggest that this subtype underwent limited evolutionary change and retained relative antigenic stability. Since its emergence in the human population during the 1968 pandemic, A(H3N2) has established itself as one of the predominant subtypes of seasonal influenza. Persistent antigenic drift in A(H3N2), together with changes in HA glycosylation and receptor-binding properties, has contributed to marked genetic divergence and variable antigenic matching with vaccine strains [31]. In China, circulating A(H3N2) viruses remained more genetically distant from the corresponding vaccine strains than viruses of the other subtypes. Frequent amino acid substitutions were identified at several residues in HA1 antigenic sites A and B, and the egg-based A(H3N2) group showed the highest dN/dS ratio. These findings suggest that A(H3N2), especially the egg-based A(H3N2) strains, had relatively greater nonsynonymous substitution pressure and antigenic variability than the other subtypes or lineages, although the dN/dS values do not indicate strong positive selection at the gene level.

More broadly, global studies on influenza evolution indicate that A(H3N2) undergoes the most rapid antigenic drift, shows the highest migration frequency, and persists for the shortest time within individual regions. In contrast, B/Victoria evolves more slowly in antigenic terms but retains substantial genetic diversity, whereas A(H1N1)pdm09 and B/Yamagata remain more evolutionarily conserved. Notably, no confirmed circulation of the B/Yamagata lineage has been reported globally since 2020, although whether this lineage has become extinct remains under investigation [16]. These lineage-specific evolutionary characteristics may underlie the observed differences in vaccine matching across seasonal influenza subtypes and lineages [4]. In this study, the genetic distance of the B/Victoria lineage remained around 2% throughout the study period. Frequent amino acid substitutions at residues 162 and 165 in the 160-loop and at residue 197 in the 190-helix may have contributed to the instability of vaccine matching in this lineage. In contrast, the B/Yamagata lineage exhibited a remarkably low overall frequency of amino acid substitutions, with most antigenic sites remaining highly conserved and genetic distance remaining stable, which may have supported its consistently high match rate with the corresponding vaccine strains over time. In summary, antigenic mismatch in A(H3N2) and the B/Victoria lineage highlights the difficulty of annual vaccine strain selection and the need for continuous antigenic and genetic surveillance.

In addition to differences in evolutionary rate among subtypes and lineages, it is also key to consider the effect of vaccine production platforms on viral antigenicity in determining vaccine match. In the present study, cell-based vaccine reference strains demonstrated higher antigenic match rates than egg-based vaccine reference strains for A(H3N2) viruses circulating in China during the 2015–2025 seasons. A retrospective analysis of global influenza surveillance data from 2002 to 2018 demonstrated that antigenic match rates for egg-based A(H3N2) vaccine strains fell below 25% in 55% of influenza seasons, compared with only 4% of seasons for cell-based strains. In seasons with high A(H3N2) activity, cell-based strains achieved match rates of 76–100%, further supporting their antigenic advantage over egg-based strains [32]. Evidence from the 2022–2023 A(H3N2)-predominant season in the United States also showed 7.7% greater protection for cell-based vaccines than for egg-based vaccines, suggesting potential clinical relevance of improved antigenic match [33]. This difference may be attributable to egg-adaptive changes that arise during propagation in embryonated eggs. Adaptive substitutions at HA antigenic sites can be introduced during egg passage, such as T160K and L194P in A(H3N2) [34]. Notably, T160K has been shown to significantly reduce neutralizing antibody titers against circulating A(H3N2) strains after vaccination [35]. This interpretation was corroborated by our sequence analysis, which showed that substitutions at HA antigenic site 160 were more frequent in the egg-based A(H3N2) than in the cell-based A(H3N2) strains. Such adaptive mutations can be largely avoided during propagation in MDCK cells, which helps preserve native viral antigenicity and improve antigenic matching between vaccine strains and circulating strains. However, the present study evaluated antigenic concordance and sequence variation rather than vaccine effectiveness, immunogenicity, or clinical protection. Therefore, these findings support further consideration of cell-based vaccine platforms in seasons with high A(H3N2) activity, but additional effectiveness and immunogenicity studies are needed to confirm their clinical benefit.

Unlike vaccine matching, NAI resistance was observed only in the A(H1N1)pdm09 subtype among seasonal influenza viruses circulating in China. Phenotypic surveillance further indicated an upward trend after resistance was first reported in 2023. However, the resistance-associated mutation H274Y was already present in sequence data in 2015 and remained at a relatively high frequency of 10–20% during 2015–2018. Although these genotype-based and phenotypic data were derived from different surveillance datasets and were not directly comparable at the individual-virus level, the early presence and relatively high frequency of H274Y may still serve as a genomic indicator of potential oseltamivir resistance. Because individual virus-level linkage was unavailable, we could not determine whether H274Y-positive viruses underwent phenotypic susceptibility testing or whether all phenotypically resistant viruses carried H274Y. H274Y mutation without permissive compensatory substitutions may be difficult to maintain stable phenotypic resistance, although the mutation reduces NA surface expression and compromises viral fitness. Phenotypically detectable resistance is more likely to persist when viral function is restored by permissive or compensatory substitutions [36]. Compensatory substitutions in the HA protein gene may also restore the functional balance between HA and NA, thereby further mitigating fitness defects associated with H274Y and contributing to the discordance between genotypic and phenotypic resistance [37]. However, compensatory or permissive mutations were not comprehensively investigated in this study. Taken together, these mechanisms may partly explain the relatively high frequency of H274Y despite the rarity of phenotypic resistance, although paired phenotypic and genomic data are needed for further confirmation. Neuraminidase inhibitor resistance may also affect viral transmissibility. Although most resistance-associated substitutions reduce transmissibility [38,39], some preserve or even enhance transmission fitness [40]. Compensatory substitutions further complicate the transmission risk of resistant viruses.

This study has several limitations. First, antigenic match rates and neuraminidase inhibitor (NAI) resistance frequencies were calculated mainly from aggregated weekly CNIC surveillance reports rather than individual virus-level data. Therefore, potential heterogeneity in sampling location, sampling time, patient characteristics, and laboratory testing could not be fully evaluated. Second, surveillance intensity and the number of viruses characterized or tested may have varied among seasons and subtypes/lineages, which could affect the stability and comparability of annual estimates, especially when sample sizes were small. Thus, comparisons across seasons and among subtypes or lineages should be interpreted with caution. Despite these limitations, CNIC weekly reports provide standardized national surveillance data and remain valuable for evaluating long-term changes in vaccine antigenic match and NAI resistance in China.

## 5. Conclusions

This study systematically evaluated the antigenic concordance between seasonal influenza vaccine strains and circulating strains in China from 2015 to 2025, as well as the emergence of neuraminidase inhibitor (NAI) resistance. Antigenic concordance for A(H3N2) and B/Victoria viruses showed marked year-to-year fluctuations. Compared with egg-based A(H3N2) vaccine reference strains, cell-based vaccine reference strains showed higher antigenic concordance with circulating strains, suggesting that vaccine production platforms should be further considered in A(H3N2)-predominant seasons. In addition, phenotypic NAI resistance has been detected in A(H1N1)pdm09 viruses circulating in China since 2023, whereas resistance-associated NA substitutions, particularly H274Y, had appeared earlier at the sequence level. These findings suggest the urgent need for the development of antigenically matched or broad-spectrum influenza vaccines, as well as highlight the importance of continuous antigenic, genetic, and antiviral resistance surveillance for optimizing vaccine strain selection and improving influenza prevention and control strategies.

## Data Availability

No new data were generated in this study. The antigenic characterization and antiviral susceptibility data were obtained from publicly available CNIC weekly influenza surveillance reports, and HA/NA sequence data were obtained from GISAID.

## Supplementary Materials

The following supporting information can be downloaded at: https://www.mdpi.com/article/10.3390/vaccines14070586/s1. Table S1. Weekly antigenic characterization and vaccine matching data for seasonal influenza viruses in China, 2015–2025; Table S2. Number of HA and NA sequences included after quality filtering by influenza season and subtype/lineage.
